# Evidence-based effect size estimation:An illustration using the case of acupuncture for cancer-related fatigue

**DOI:** 10.1186/1472-6882-9-1

**Published:** 2009-01-13

**Authors:** Michael F Johnston, Ron D Hays, Ka-Kit Hui

**Affiliations:** 1 Center for East-West Medicine, Department of Medicine, David Geffen School of Medicine, University of California, Los Ángeles, California, USA; 2 Department of Medicine, David Geffen School of Medicine, University of California, Los Ángeles, California, USA

## Abstract

**Background:**

Estimating a realistic effect size is an important issue in the planning of clinical studies of complementary and alternative medicine therapies. When a minimally important difference is not available, researchers may estimate effect size using the published literature. This evidence-based effect size estimation may be used to produce a range of empirically-informed effect size and consequent sample size estimates. We provide an illustration of deriving plausible effect size ranges for a study of acupuncture in the relief of post-chemotherapy fatigue in breast cancer patients.

**Methods:**

A PubMed search identified three uncontrolled studies reporting the effect of acupuncture in relieving fatigue. A separate search identified five randomized controlled trials (RCTs) with a wait-list control of breast cancer patients receiving standard care that reported data on fatigue. We use these published data to produce best, average, and worst-case effect size estimates and related sample size estimates for a trial of acupuncture in the relief of cancer-related fatigue relative to a wait-list control receiving standard care.

**Results:**

Use of evidence-based effect size estimation to calculate sample size requirements for a study of acupuncture in relieving fatigue in breast cancer survivors relative to a wait-list control receiving standard care suggests that an adequately-powered phase III randomized controlled trial comprised of two arms would require at least 101 subjects (52 per arm) if a strong effect is assumed for acupuncture and 235 (118 per arm) if a moderate effect is assumed.

**Conclusion:**

Evidence-based effect size estimation helps justify assumptions in light of empirical evidence and can lead to more realistic sample size calculations, an outcome that would be of great benefit for the field of complementary and alternative medicine.

## Background

In their content analysis of all scientific reviews of grant applications submitted by the Community Clinical Oncology Program for clinical trials of complementary and alternative medicine (CAM) to the National Cancer Institute, Buchanan and colleagues (p. 6685) indicated that one of the five major concerns raised by reviewers has been "justifying the anticipated effect sizes used to determine sample size."[1] With regards to published research studies, meta-analytic and systematic reviews regularly point to sample size as a troubling issue.[2] Often, the treatment effects are positive and non-trivial in size, but results are non-significant due to insufficient sample size. This recurrent finding suggests that when considering and planning studies, CAM researchers may tend to over-estimate expected treatment effect sizes.

Estimating effect size without data from a randomized controlled trial (RCT) is an issue faced by researchers in general. An approach that is often preferred is to power the study to detect a minimally important difference (MID)–the magnitude of improvement for which patients would consider a course of treatment to be worthwhile.[3] In some instances, an MID estimate may be unavailable so a pilot study may be needed. When it is neither possible to specify an MID nor feasible to collect pilot data, researchers can make use of the published literature to derive plausible effect size estimates.

We propose a four-step process of *evidence-based effect size estimation*, especially for situations when there is evidence from pre-post studies but little or no evidence from randomized controlled trials. The first step is to draw upon published literature to specify a range of assumptions about (a) the amount of change in the treatment group and (b) the amount of change in the control group. The second step is to make a plausible assumption about the amount of change in the treatment group relative to control based upon evidence gathered in step one. The third step is to carry out a power analysis to estimate sample size. The fourth step is to refine assumptions about effect size. We illustrate evidence-based effect sized estimation for a newly developing area of interest, acupuncture in the treatment of fatigue in patients who have completed primary treatment for cancer.

## Methods

Suppose that we plan to carry out a clinical trial and that individuals with breast cancer will be randomly assigned to either treatment (acupuncture) or control. Assume further that the primary outcome of interest is continuous rather than categorical. The preferred model of analysis in this situation is the analysis of co-variance (ANCOVA) because it controls for baseline imbalance and increases the statistical power to detect a difference.[4,5]

### Step 1: Reviewing the literature

#### Part A. Estimating effect of acupuncture in relieving fatigue

We carried out a systematic literature search in PubMed to identify studies of acupuncture for relief of fatigue in breast cancer survivors. In our initial search wave, we used key words "breast cancer" in combination with "acupuncture" and "fatigue" and then "breast cancer" with "acupuncture". We further searched the reference lists of identified articles and then also used Google Scholar to search the web. From the approximately 125 studies we examined, only one met our three search criteria: (1) manual acupuncture, (2) fatigue as a measured outcome, and (3) breast cancer survivors as the target population. This published study was an uncontrolled, Phase II study that involved provision of acupuncture to fatigued breast cancer survivors over the course of approximately 8 weeks.[6] In a second search wave, we looked for studies examining the effect of acupuncture in relieving fatigue in persons without breast cancer. This search identified two additional studies (see Table 1).[7,8]

**Table 1:** Information extracted from utilized studies concerning recovery from fatigue

**Acupuncture Studies (Treatment)**								
Study ID	Patient Population	Instruments, Units	*n*^1^	$\bar{x}$_*base *_^2^	*S*_*base *_^3^	$\bar{x}$_*f*-*up *_^4^	$\bar{x}$_*f*-*up *_^5^	Cohen's D

Vickers et al.	Breast cancer survivors	Brief Fatigue Inventory	31	6.47	1.21	4.55	2.16	1.02

VickersSMLG			31	6.47	1.21	4.97	2.16	0.80

Vickers90%			31	6.47	1.21	5.21	2.16	0.62

A-W.AVG			199					0.56

Hays et al.	Consecutive patients	SF-36 vitality/energy (reversed)	54	57.00	9.60	52.00	8.90	0.54

Harris et al.	Patients with fibromyalgia	Multi-dimen'l fatigue inventory	114	16.60	3.19	14.98	3.89	0.45

**Wait-list Control Groups**								

Carpenter	Breast cancer survivors	Fatigue Scale from FACT	16	5.82	5.00	3.99	5.00	0.37

Courneya	Breast cancer survivors	Multidimensional Fatigue Inventory	28	10.80	8.80	8.80	8.10	0.24

Stanton	Breast cancer survivors	Linear analogue scale for fatigue	136	44.00	19.90	40.16	18.40	0.20

C-W.AVG			246					0.16

Pinto	Breast cancer survivors	POMS S. Form Fatigue Subscale	43	41.66	25.04	42.28	26.20	0.02

Badger	Breast cancer survivors	SF 36 (reversed)	24	37.63	25.50	37.15	28.20	-0.02

1. number of observations.

2. mean at baseline.

3. standard deviation at baseline.

4. mean at follow-up.

5. standard deviation at follow-up.

The three studies referenced in Table 1 all assessed change in fatigue. Each study shows acupuncture to have a beneficial effect in relieving fatigue and each use different measurements and patient populations. The Vickers study used the nine-item Brief Fatigue Inventory (BFI), for which higher scores reflect greater fatigue. The Harris study employed the Multidimensional Fatigue Inventory (MFI), which is also constructed so that higher scores reflect greater fatigue. In contrast, the Hays et al. study used the SF-36. In the SF 36, lower scores reflect greater fatigue; for the sake of consistency with the BFI and the MFI, we reverse the SF 36 scoring. One very important difference between the three studies is that they were carried out with different patient populations: breast cancer survivors (Vickers), patients with fibromyalgia (Harris), and consecutive patients from a clinic (Hays et al.). It may be the case that acupuncture is more or less effective in relieving fatigue in these different populations.

To compare the effect of acupuncture in relieving fatigue, we used G*Power 3 software[9,10] to calculate, on a study by study basis, Cohen's D:

(1) $$\text{Cohen's D}=\frac{\left|mean_{1}-mean_{2}\right|}{S.D.},$$

where *mean*_1 _and *mean*_2 _are the means at pre and post measurements. The S.D. (standard deviation) is weighted to take account of the standard deviation for the first and the second mean and the third term with the correlation (*ρ*) is present because we assume that these two standard deviations are nonindependent:

(2) $$\;\sigma_{\bar{X}1-\bar{X}2}=\sqrt{\sigma_{\bar{X}1}^{2}+\sigma_{\bar{X}2}^{2}-2*\rho *\sigma_{\bar{X}1}*\sigma_{\bar{X}2}}$$

Standard deviations may vary from pre to post; by accounting for both, the resulting assumption is more robust. For the power analysis, we used the following specifications: a Type I error rate of *α *= .05, statistical power of .80, a two-tailed test, and a rather conservative correlation between pre and post measurements of 0.5. It could be argued that a correlation of 0.7 is a more conventional default value and if this were used, the resulting Cohen's D would be higher (indicating a stronger effect size). Since we are looking at the change from baseline to follow-up within the same participants, the analytical model we specify is a t-test for dependent means. The standards given by Cohen to interpret D is that a large effect size is one that is equal to or greater than 0.80, a medium effect is one that is equal to or greater than 0.50 but less than 0.80, and a small effect is one that is equal to or greater than 0.20 but less than 0.50.

Whereas the Vickers study yielded a large effect size, the other two yielded small to medium effects. It is not surprising that the Hays and Harris studies have a lower Cohen's D than that reported in the Vickers study. The Vickers study was designed specifically to study the effects of acupuncture upon fatigue; they did so by only including patients with greater than mild fatigue. In contrast, the Harris and Hays studies did not specify exclusion or inclusion criteria related to fatigue. Since patients in these studies will include people with low levels of fatigue that have little room for improvement, the overall mean improvement is lower.

Although the effect of acupuncture upon cancer-related fatigue was large in the Vickers study, Price and colleagues[11] argued that if patients were to experience acupuncture according to the philosophy of Chinese medicine (individualized treatment) they would achieve a more complete relief of fatigue than those who experience acupuncture according to the fixed-point standard prescription used in that study. The three uncontrolled studies were performed on different populations, they used different treatment protocols and, presumably, practitioners with different levels of skill and/or experience performed them. The weighted average approach assumes, essentially, that these differences have minimal effects upon the clinical results. If there were reason to treat one study as more predictive for the planned trial, a more complicated weighting scheme could be developed. Similarly, more emphasis could be placed on that trial, such as we have done in developing alternative estimates for the Vickers trial, namely VickersSMLG and Vickers90% (see below).

In our subsequent sections, we consider the effect of acupuncture in relieving cancer-related fatigue from a variety of perspectives. First, we consider whether the effect reported by Vickers (Cohen's D = 1.02) is representative of what would generally occur in a population of breast cancer survivors. In another approach we combine the information from the three studies via a weighted average, which yields a Cohen's D of 0.56. In a third approach, we adjust the follow-up mean reported by Vickers downwards so that the Cohen's D is capped at the smallest value in the large effect size range specified by Cohen (i.e., 0.80, which yields a follow-up mean of 4.97). A fourth approach is to argue that the Vickers study captured a true effect but that the scores at follow-up may have been unusually large. That is to say, we consider the mean reported by Vickers at follow-up within the context of a 90% confidence interval and take the lower bound (a follow-up mean of 5.21). We refer to these approaches, respectively as: Vickers, A-W.AVG, VickersSMLG, and Vickers90%. The Cohen's D for each is reported in Table 1.

#### Part B. Estimating effect of participating in a wait-list control group receiving usual care

Birch, in a review of placebo effects in acupuncture research, advocates for a distinction between trials that involve an inert placebo (such as wait list controls) and those that involve non-inert treatments such as sham acupuncture.[12] Though they do not use the terminology of inert placebos, Walach and Jonas point out that such groups account for the beneficial effect of participation in a clinical trial that is due to natural history, spontaneous remission, and regression to the mean.[13] The use of non-inert placebos, such as sham acupuncture, are known to produce an effect that is stronger than that of an inert placebo.[12] There are several hypotheses regarding why but as of yet none are strongly supported by the data and agreed upon by a wide number of experts. This is not a concern for the issue at hand because we use the published literature to estimate an effect for an inert placebo by identifying wait-list controls receiving standard care.

To identify studies that investigated the effect of an inert placebo in relieving fatigue in breast cancer patients, we again carried out a systematic search of PubMed, this time using "breast cancer" and "fatigue" with the following limits: English and Randomized Controlled Trial. Again, the search yielded approximately 200 hits, of which we identified five[14-18] (see Table 1) that met our criteria: (1) a randomized controlled trial with fatigue as a measured outcome, (2) a wait-list control group receiving usual care, and (3) target breast cancer survivors. In addition, we hand-searched reference lists of identified articles, identified systematic reviews of similar topics, and then also used Google Scholar to search the web.

At first glance, it might appear inappropriate to use non-acupuncture trials from fatigue studies to extrapolate power-relevant information. The key point is that these control arms are wait-list controls. The logic of using this information is that there will be no appreciable systematic difference in the amount of change experienced by wait-list control groups in an acupuncture trial and that experienced by wait-list control groups in, for example, exercise trials.

We calculated a Cohen's D for each trial. Notice that in four out of the five trials, breast cancer patients who received an inert placebo, as a group, showed a recovery in fatigue. To summarize the information from the five wait-list control groups receiving standard care, we carried out a weighted average (C-W.AVG), with the resultant pooled Cohen's D estimated to be 0.16.

### Step 2: Estimating an Inclusive effect size

Using the published literature to estimate the effect of acupuncture upon relieving fatigue relative to a wait-list control receiving standard care is based upon the assumption that the patient population employed by Vickers is comparable to those in the controlled studies because they all include breast cancer survivors. However, the Vickers study had in place inclusion/exclusion criteria that bounded this population to a subset of people with moderate or high fatigue, while the controlled studies did not have in place these criteria. The potential problem is that moderate and highly fatigued breast cancer survivors may experience regression to the mean at a higher rate than those who are only mildly fatigued. While this explanation cannot be ruled out, it is the case that epidemiological and related studies make the point that fatigue is one of the most *persistent *problems facing cancer survivors.[19,20] Without any evidential basis to assume that breast cancer survivors with moderate and high amounts of fatigue would be more likely than others to progress towards recovery quickly, we assume that the effects for the general population of breast cancer survivors as captured in the five studies above are also representative of the subset of breast cancer survivors with moderate and high levels of fatigue.

In Table 2, we display a series of comparisons between treatment and control. We chose these comparisons to provide a sense of the range of potential effect sizes, as well as to offer some insight into the consequences of particular assumptions that underlay the calculation of effect size. We compute an effect size for the comparison between Vickers and Badger. The effect size for the Badger study and the result is slightly negative because, on average, participants in the control group reported more fatigue at study conclusion than at study beginning. Since the floor for Cohen's D is zero, we set Cohen's D = 0 for the Badger study. Comparing Vickers and Badger yields the largest effect size. Of all the effect size calculations for treatment that involve the Vickers study, the weighted average has the lowest effect. Of all the effect size calculations for control, the one reported by Carpenter is the largest and we see this as representing an upper bound for the amount of improvement a control group could show in the context of an RCT. To produce a conservative estimate of Cohen's D between treatment and control, we therefore compare the weighted average for acupuncture to Carpenter's control group. To further our sense of the way that stochastic processes may influence the realized effect size, we compare Vickers90 to first Carpenter and then the weighted average of control group studies.

**Table 2:** Effect and sample size calculations

**Treatment Group**	**Control Group**	**Treatment Effect Size**	**Control Effect Size**	**Comparison Effect Size**	**R**^2^	**Effect Size f**^2^	**Sample Size**
Vickers	Badger	1.02	0.00	1.02	0.21	0.27	40

VickersSMLG	C-W.AVG	0.80	0.16	0.64	0.09	0.10	101

Vickers90%	C-W.AVG	0.62	0.16	0.46	0.05	0.05	187

A-W.AVG	C-W.AVG	0.56	0.16	0.40	0.04	0.04	235

Vickers90%	Carpenter	0.62	0.37	0.25	0.02	0.02	476

A-W.AVG	Carpenter	0.56	0.37	0.19	0.01	0.01	957

**Figure 1:**
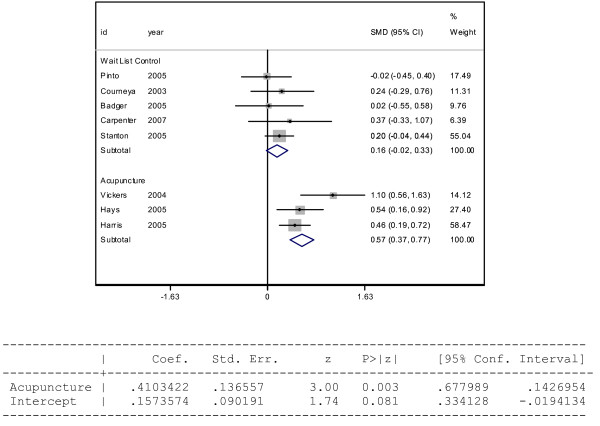
**Meta-analytic results of fatigue recovery**. Acupuncture vs. breast cancer survivor wait list controls.

A final method we employ is to compare the two weighted averages. This method relates very closely to a random effects meta-analysis[21] which treats each study as a unit. Figure 1 displays a forest plot of the meta-analysis, with the 5 wait-list controlled studies in the upper half of the plot and the three acupuncture studies in the lower half of the plot. The overall effect for the controls (0.16) is equal to the weighted average of the controls. Similarly, the overall effect for the acupuncture studies (0.57) is equal to the weighted average for the acupuncture studies (within rounding error). Is the effect of acupuncture in relieving fatigue attributable to chance or is there a treatment effect? One of the strengths of the random effects meta-analysis is that each study can be defined by a covariate such as average duration of follow-up, a measure of study quality, or a measure of geographic location of study and a formal statistical test can be carried out on that covariate. For our example, the covariate of interest is whether participants in the study received acupuncture or standard care. The p-value (0.003) indicates that subjects who participated in a study that involved administration of acupuncture experienced a significantly higher amount of fatigue relief than those subjects who participated in a study as a member of a control group receiving standard care.

The values of Cohen D displayed in Table 2 illustrate that the effect of acupuncture in relieving fatigue among breast cancer survivors relative to a wait-list control receiving standard care varies from very large (1.02) to very small (0.11). If it is assumed that acupuncture produces an extremely large effect, as reported by Vickers, and that patients in the wait-list control will not improve (Badger), one would conclude that acupuncture has a large effect upon relieving cancer-related fatigue (Cohen's D = 1.02). If it is assumed that people in the control group will recover at a rate equal to the average of the controls (A-W.AVG) and that the effect for acupuncture is the smallest large effect (VickersSMLG), one would conclude that the difference between the groups is moderate (hovering around 0.50). If we continue to assume that the effect of acupuncture is at the level of the smallest large effect, but that the control group will evidence a relatively great deal of recovery, as was the case in the Carpenter study, then one would conclude that acupuncture produces a somewhat small effect in relieving cancer-related fatigue in breast cancer survivors (Cohen's D of approximately 0.20).

## Results

### Step 3: Power Analysis

To carry out a power analysis for an ANCOVA with G*Power 3, we begin with a baseline model that does not include a coefficient for the treatment effect:

(3) *y*_*i *_= *β*_0 _+ *β*_1_*X*_1 _+ *e*_*i*_.

In model (3), *y*_*i *_is the amount of fatigue each participant reports at completion of the treatment. The coefficient *β*_0 _is a constant, representing the mean amount of fatigue in the control group at completion, given a score of zero fatigue at baseline. The coefficient *β*_1 _represents the scaled contribution of fatigue at baseline on fatigue at completion. The parameter *e*_*ij *_represents the difference between an individual's predicted change in fatigue and actual change in fatigue.

To specify the power analysis, we assume a Type I error rate of *α *= .05 and statistical power of .80. There are two predictors in the original model, so there are 2 degrees of freedom in the numerator and we add one predictor so that number of predictors is specified as 1. We also need to specify how much the R^2 ^will increase when this one predictor for treatment effect is included into the model. This requires a transformation from Cohen's D to R^2^, for which we use a freely available effect size calculator.[22] Information for the power calculation is found in the R^2 ^column of Table 2.

We power this equation with respect to an R^2 ^that is statistically different from a model with *β*_0 _and *β*_1_. In doing so, we assume that one additional parameter will be added to the model, *β*_2_, which is associated with the independent variable of interest: treatment effect. The coefficient *β*_2 _represents the mean amount of fatigue at completion amongst people who received acupuncture (the treatment), holding constant fatigue at baseline (*β*_1_).

The final column of Table 2 shows that the sample size needed for the ANCOVA model described above (1) in light of assumptions about effect size is very large, ranging from 40 (20 per arm) to 957 (479 per arm). If a very narrow range resulted from step 3, this would be a potential stopping point. In most cases, however, we would expect that researchers would benefit from moving on to step 4.

### Step 4: Refine Assumptions

The smallest sample size (n = 40; 20 per arm) is required when it is assumed that acupuncture produces an extraordinary result and that the control group does not recover at all, as was the case in the Badger study. When it is assumed that acupuncture produces the smallest large effect and that the control group recovers at a rate equal to the average across all control studies, than the sample size requirements are larger (n = 101; 52 per arm), but very feasible. If we assume that acupuncture has a moderate effect, as is associated with Vickers90%, and that the control group recovers at a rate equal to the average across all control studies, then the sample size requirements double to n = 187 (94 per arm). If we continue to assume that the control group recovers at an average rate but that the effect of acupuncture is only moderate (average from all studies), the sample size requirements are yet higher (n = 235; 118 per arm).

It seems unrealistic to assume that on average participants in the control group will not recover at all (Badger) or even that there will be minimal recovery (Pinto). Although it is conceivable that an acupuncture arm will produce results just as strong as those in the Vickers study, it is also plausible that it might be a little less. At this point, the research team would take stock of their resources and goals in light of the assumptions they consider to be most reasonable. If they want to proceed with a phase III type trial, it would seem that at least 101 subjects (52 per arm) would be required. Is this feasible? If they decide to pursue a trial along the lines of a phase IIB type, then they would want to adjust their expectations in terms of results.

### Epilogue

Molassiotis and colleagues[23] recently published a pilot randomized controlled trial that provides a valuable opportunity to compare the effect sizes we estimated from uncontrolled data to randomized controlled trial data. Their study involves 47 patients randomizes to either an acupuncture group (n = 15), an acupressure group (n = 16) or a sham acupuncture group (n = 16). The 20-minute acupuncture session consisted of inserting needles into three points associated with energy (LI4, SP6, and ST36) bilaterally three times a week over the course of 2 weeks. The sham acupressure group was taught to apply pressure to three points not associated with energy (LI12, GB33 and BL61). Researchers collected information about fatigue with the Multidimensional Fatigue Inventory (MFI) at baseline, end of the 2-week treatment, and follow-up (2 weeks after completing treatments). We extract relevant information from this study and compute Cohen's D effect size (see Table 3).

**Table 3:** Information extracted from randomized controlled pilot study involving acupuncture for fatigue

**Acupuncture Studies (Treatment)**								
**Study ID**	**Patient Population**	**Instruments, Units**	***n *(1)**	$\bar{x}$**_*base *_(2)**	***S*_*base *_(3)**	$\bar{x}$_*f*-*up*_**(4)**	*S*_*f*-*up *_**(5)**	**Cohen's D**

Moulassiotis, Baseline to Completion	Cancer survivors, primarily lymphoma and breast	Multidimensional Fatigue Inventory	13	16.4	2.40	10.5	3.00	2.15

Moulassiotis, Baseline to Follow-up	Cancer survivors, primarily lymphoma and breast	Multidimensional Fatigue Inventory	13	16.4	2.40	12.8	3.20	1.25

**Wait-list Control Groups**								

Moulassiotis, Baseline to Completion	Cancer survivors, primarily lymphoma and breast	Multidimensional Fatigue Inventory	13	17.8	2.50	17.7	2.60	0.04

Moulassiotis, Baseline to Follow-up	Cancer survivors, primarily lymphoma and breast	Multidimensional Fatigue Inventory	13	17.8	2.50	16.9	3.00	0.32

6. number of observations.

7. mean at baseline.

8. standard deviation at baseline.

9. mean at follow-up.

10. standard deviation at follow-up.

Results from the Molassiotis study suggest a large effect for the acupuncture group and has ambiguous implications for the control group. At the conclusion of the two week treatment, patients in the Molassiotis acupuncture group reported a tremendous improvement in fatigue (Cohen's D of 2.15) and at the end of the follow-up period reported a large improvement in fatigue relative to baseline (Cohen's D of 1.25). The amount of recovery exhibited by the Molassiotis control group essentially spans the full range presented in the wait-list control groups (Table 1), with a Cohen's D of 0.04 at the conclusion of the two week treatment and a Cohen's D of 0.32 at the end of the follow-up period.

The Molassiotis results, given that they come from a randomized controlled trial, merit primary consideration in estimating an effect size. But there is substantial variability in means (and standard deviations) across the three time periods. In situations with such variability, information from the observational studies may be used to finalize assumptions. In the instance of acupuncture for fatigue, one conservative approach would be to use the effect size reported from the follow-up for treatment (Cohen's D of 1.25) and the effect size from the follow-up for the control (Cohen's D of 0.32), which yields a comparison effect size of 1.25 – 0.32 = 0.93.

## Discussion

Specifying anticipated effect size of treatment relative to control requires assumptions. To justify these assumptions, we propose evidence-based effect size estimation: anchoring assumptions in the published literature as much as possible, preferably to results from randomized controlled trials. It may be that at least some researchers have employed a similar process in grant applications and would therefore see nothing novel about evidence-based effect size estimation. Nonetheless, it is important to make this process explicit. One reason to do so is because effect sizes derived from published literature may exhibit a considerable amount of variation. In such situations, researchers will need to discern the key factors that produce variability across the studies. Some examples that involve patients include differences in the length of illness, severity of illness, and comorbidities. Clinic-based examples include differences in clinician experience and skill, treatment length, and treatment regiment. We are hopeful that a formal conversation about these processes will produce a consensus about the best possible standard approach.

The studies we employed to estimate the effect size of the control group reinforce the lesson that when there are a small number of participants, results may be unstable. The Badger study, with 24 participants, shows there to be no improvement in fatigue in the control group, but the Carpenter study, with 16 participants, shows there to be considerable improvement. When the control group is comprised of only a small number of participants, improvement by just a few people may effectuate a noticeable improvement for the control group as a whole. Importantly, improvement may be due to control group participants taking advantage of the therapy from other sources ("drop-ins")[24]^25 ^or more simply to their own natural healing systems becoming activated in response to participating in a clinical trial (the Hawthorne effect). This uncertainty points to the need for keeping track of drop-ins, both for the sake of clarifying which process may be operating to effectuate recovery in the control group as well as for purposes of statistical analyses. Disentangling these effects may provide useful clues as to the potential usefulness of acupuncture for those carrying out meta-analyses summarizing studies with mixed results.

## Conclusion

Evidence-based effect size estimation leads to elaborating assumptions in light of empirical evidence and showing the extent to which assumptions are robust. Our illustration with acupuncture for cancer-related fatigue shows that several different assumptions are plausible. For this reason, assumptions may be more open to discussion. We see this as positive because discussion would likely lead to more realistic sample size calculations – an outcome that would be of great benefit for the field of complementary and alternative medicine.

## Competing interests

The authors declare that they have no competing interests.

## Authors' contributions

MJ conceptualized the study, its design and coordination, drafted and revised the manuscript, and performed statistical analyses. RH participated in study design, review of statistics, and manuscript revision. KH participated in study conceptualization and manuscript revision.

## Pre-publication history

The pre-publication history for this paper can be accessed here:


